# Analysis of silica dust monitoring results and prevention implications in industrial and mining enterprises of Sichuan Province, China (2021–2025)

**DOI:** 10.3389/fpubh.2026.1852867

**Published:** 2026-06-05

**Authors:** Weizhong Chu, Xiangxiang Lu, Weiwei Shang, Leping Qiu

**Affiliations:** Sichuan Center for Disease Control and Prevention, Chengdu, Sichuan, China

**Keywords:** compliance rate, free silica content, occupational health monitoring, occupational silica exposure, silica dust

## Abstract

**Objectives:**

To analyze the status and temporal trends of silica dust hazards in industrial and mining enterprises in Sichuan Province from 2021 to 2025, and to identify key predictors of non-compliance to support evidence-based prevention strategies.

**Methods:**

A 5-year retrospective monitoring study was conducted among 1,200 enterprise monitoring cases across four key industries. A total of 3,669 posts were included. Respirable dust concentrations and compliance rates were analyzed using descriptive statistics, chi-square test, trend tests, rank-sum test, and binary logistic regression.

**Results:**

The overall compliance rate for silica dust posts was 82.2%. Compliance improved significantly from 2021 to 2025 (*p* < 0.05) but was lower in larger enterprises and at posts with higher free silica content (both *p* < 0.05). The Northwest Sichuan region and machinery industry showed the lowest compliance. Logistic regression confirmed that year, industry, economic region, enterprise scale, and free silica content were independent predictors of compliance.

**Conclusion:**

Although overall compliance improved, high-risk areas remain in large enterprises, machinery and building materials industries, high free silica operations, and underdeveloped regions. Targeted risk-based interventions are urgently needed.

## Introduction

Silica dust remains one of the most prevalent and severe occupational hazards globally, particularly in low- and middle-income countries where industrialization continues to expand (1, 2). Long-term inhalation of respirable crystalline silica (RCS) leads to silicosis, an irreversible and progressive fibrotic lung disease that imposes a substantial public health burden (3, 4). In China, despite intensified regulatory efforts under the Law on Prevention and Control of Occupational Diseases, silicosis remains the most reported occupational disease, with non-coal mines and specific manufacturing sectors exhibiting persistently high exposure risks (5, 6). Recent risk assessments indicate that without effective intervention, cumulative incidence rates of silicosis could reach alarming levels over the next few decades.

Sichuan Province, a major industrial hub in western China characterized by rich mineral resources and diverse manufacturing bases, faces distinct challenges in silica dust hazard control. The province’s economic landscape includes extensive mining, metallurgy, building materials, and machinery industries, all of which are primary sources of silica dust (7). Although provincial authorities have implemented special governance actions and strengthened occupational health surveillance, the effectiveness of these interventions and the evolving patterns of silica dust hazards remain to be rigorously assessed. Previous studies have highlighted significant disparities in exposure levels based on industry type, enterprise scale, and geographical location, yet comprehensive longitudinal data analyzing these factors simultaneously remain limited (8, 9).

Understanding the spatiotemporal evolution of silica dust hazards is crucial for optimizing resource allocation and developing targeted prevention strategies. Global evidence suggests that while engineering controls and regulatory enforcement can reduce exposure, emerging risks in small-scale operations and specific high-silica processes necessitate a shift towards targeted risk intervention (10, 11). Furthermore, the relationship between enterprise scale and compliance is complex; contrary to the assumption that larger enterprises are better regulated, some evidence suggests that complex processes in large facilities may pose unique control challenges (12, 13).

This study analyzes monitoring data from 2021 to 2025 covering 1,200 industrial and mining enterprises in Sichuan Province. By examining trends in compliance rates, concentration levels, and their associations with industry, region, enterprise scale, and free silica content, we aim to provide a robust evidence base for refining occupational health policies. Our findings seek to address gaps in current knowledge regarding the efficacy of existing control measures and to inform region- and industry-specific targeted risk reduction strategies for this high-burden setting.

## Methods

### Study design and population

A retrospective observational study was conducted in Sichuan Province from 2021 to 2025. Enterprises registered in the occupational disease hazard declaration system with silica dust exposure were eligible. Stratified random sampling was used to select 60 enterprises per industry annually: mining, metallurgy, building materials, and machinery, totaling 1,200 enterprise monitoring cases over 5 years.

Within each single year, enterprises were independently sampled and non-overlapping. Repeated monitoring of the same enterprise across different years was allowed. The annual repetition rate and proportion of repeated enterprises are shown in Table 1.

**Table 1:** Annual repetition and overlapping status of monitored enterprises (2021–2025).

Year	Total monitored enterprises	Repeated enterprises from previous year	Annual repetition rate (%)
2021	240	—	—
2022	240	46	19.2
2023	240	58	24.2
2024	240	69	28.8
2025	240	69	28.8
Total (5-year)	1,200	242	20.2

Repetition rate refers to the proportion of enterprises monitored repeatedly in the subsequent year; overlapping enterprises represent cross-year repeated monitoring subjects.

### Data collection and measurement

#### Region and enterprise scale

Five economic regions were defined: Chengdu Plain, Southern Sichuan, Northeastern Sichuan, Panxi, and Northwest Sichuan. Enterprise scale was classified into micro, small, medium, and large according to the Statistical Classification of Large, Medium, Small, and Micro Enterprises (2017) (14).

#### Field sampling and analysis

Area sampling was used for fixed posts; personal sampling was used for mobile posts. All sampling strictly followed GBZ 159–2004 and GBZ/T 192.1–2007 (15, 16). Respirable dust was separated using cyclone pre-separators (personal sampling) and impactor pre-separators (area sampling), conforming to ISO 7708 (BMRC B-curve). Sampling duration was 15 min with a flow rate of 20 L/min. Sampling pumps were calibrated before and after sampling; results within ±5% of the set flow were considered valid.

Free silica content in dust samples was determined using the pyrophosphate method in strict accordance with national standards.

All results reported are respirable dust concentrations. The occupational exposure limit used was the permissible concentration-time weighted average (PC-TWA) specified in GBZ 2.1–2019 (17).

#### Calculation of 8-h TWA

For personal sampling with workdays >8 h, the measured concentration was used as the shift average. For area sampling, if work duration exceeded 8 h, TWA was calculated using actual exposure time. If work duration was less than 8 h, TWA was calculated using 8 h as denominator, following GBZ 2.1–2019.

##### Quality control

All participating laboratories underwent unified training and proficiency testing. A quality assurance protocol included random data audits and on-site re-inspections of approximately 5% of the sampled enterprises to ensure data validity and representativeness.

### Statistical analysis

SPSS 30.0 was used for data sorting and statistical analysis. Medians and interquartile ranges described non-normally distributed concentrations. Chi-square (*χ*^2^) tests and Chi-square trend tests (*χ*_trend_^2^) were used for compliance rates. Binary logistic regression was performed to identify independent factors associated with non-compliance. A two-sided *p* < 0.05 was considered statistically significant.

## Results

### General characteristics

From 2021 to 2025, 1,200 enterprise monitoring cases and 3,669 posts were included. Among them, 1,774 posts were measured by area sampling and 1,895 by personal sampling. All measurements were respirable dust concentrations.

### Overall respirable dust monitoring results and temporal trends

**Figure 1:**
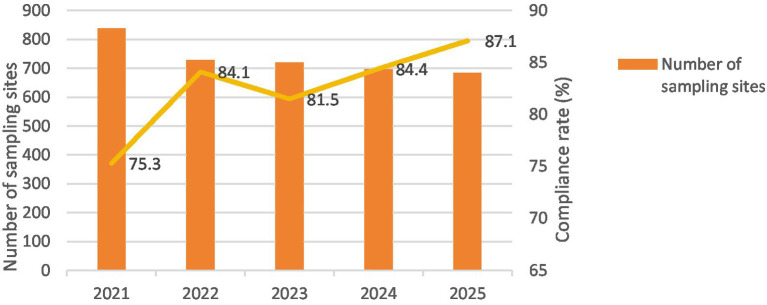
Trends in sampling sites and compliance rate of occupational dust in Sichuan Province, 2021–2025.

The overall qualified compliance rate of respirable silica dust across all monitored posts was 82.2% (3,016/3,669), with a median dust concentration of 0.32 mg/m^3^ (IQR: 0.17–0.54 mg/m^3^). Significant temporal improvements in compliance were observed during the 5-year monitoring period. The annual compliance rate increased steadily from 75.3% in 2021 to 87.1% in 2025, presenting a statistically significant upward trend (*χ*^2^ = 30.948, *p* < 0.001). In contrast, the median respirable dust concentration fluctuated slightly annually without a statistically significant linear trend (*p* > 0.05). Detailed annual monitoring data on concentration levels and compliance rates are summarized in Table 2. The temporal trend of compliance rates from 2021 to 2025 is illustrated in Figure 1.

**Table 2:** Comparison of silica dust monitoring results in industrial and mining enterprises in Sichuan Province by different characteristics, 2021–2025.

Characteristic/category	No. of posts monitored	*M*(*P*_25_, P_75_) (mg/m^3^)	No. of compliant posts	Compliance rate (%)	*χ*^2^/*χ*_trend_^2^ value*	*P*-value	*H*-value	*P*-value
Year								
2021	839	0.3(0.13,0.58)	632	75.3	30.948^a^	<0.001	6.693^b^	0.153
2022	729	0.31(0.15,0.51)	613	84.1				
2023	720	0.35(0.19,0.55)	587	81.5				
2024	697	0.33(0.18,0.51)	588	84.4				
2025	684	0.35(0.2,0.53)	596	87.1				
Economic zone								
Chengdu Plain	1703	0.31(0.15,0.5)	1,411	82.9	67.742^c^	<0.001	31.389^b^	<0.001
Southern Sichuan	598	0.37(0.19,0.6)	472	78.9				
Northeastern Sichuan	456	0.34(0.2,0.59)	377	82.7				
Panxi	682	0.32(0.19,0.47)	605	88.7				
Northwest Sichuan	230	0.38(0.15,0.88)	151	65.7				
Industry sector								
Mining	1,013	0.32(0.17,0.53)	861	85	46.398^c^	<0.001	45.196^b^	<0.001
Metallurgy	991	0.28(0.13,0.48)	860	86.8				
Building materials	781	0.33(0.17,0.6)	624	79.9				
Machinery	884	0.36(0.2,0.56)	671	75.9				
Enterprise scale								
Micro	1,216	0.32(0.18,0.53)	1,030	84.7	24.1^a^	<0.001	6.813^b^	0.078
Small	2047	0.32(0.16,0.53)	1,689	82.5				
Medium	349	0.34(0.17,0.57)	259	74.2				
Large	57	0.35(0.26,0.66)	38	66.7				
Total	3,669	0.32(0.17,0.54)	3,016	82.2				

^a^Chi-square test for trend (*χ*_trend_^2^); ^b^Kruskal–Wallis H statistic; ^c^Pearson chi-square (*χ*^2^) value.

### Stratified monitoring results by enterprise scale, economic region, and industry

Stratified analysis demonstrated significant differences in respirable silica dust exposure levels and compliance rates across enterprises of different scales, economic regions, and industrial sectors, as detailed in Table 2.

**Figure 2:**
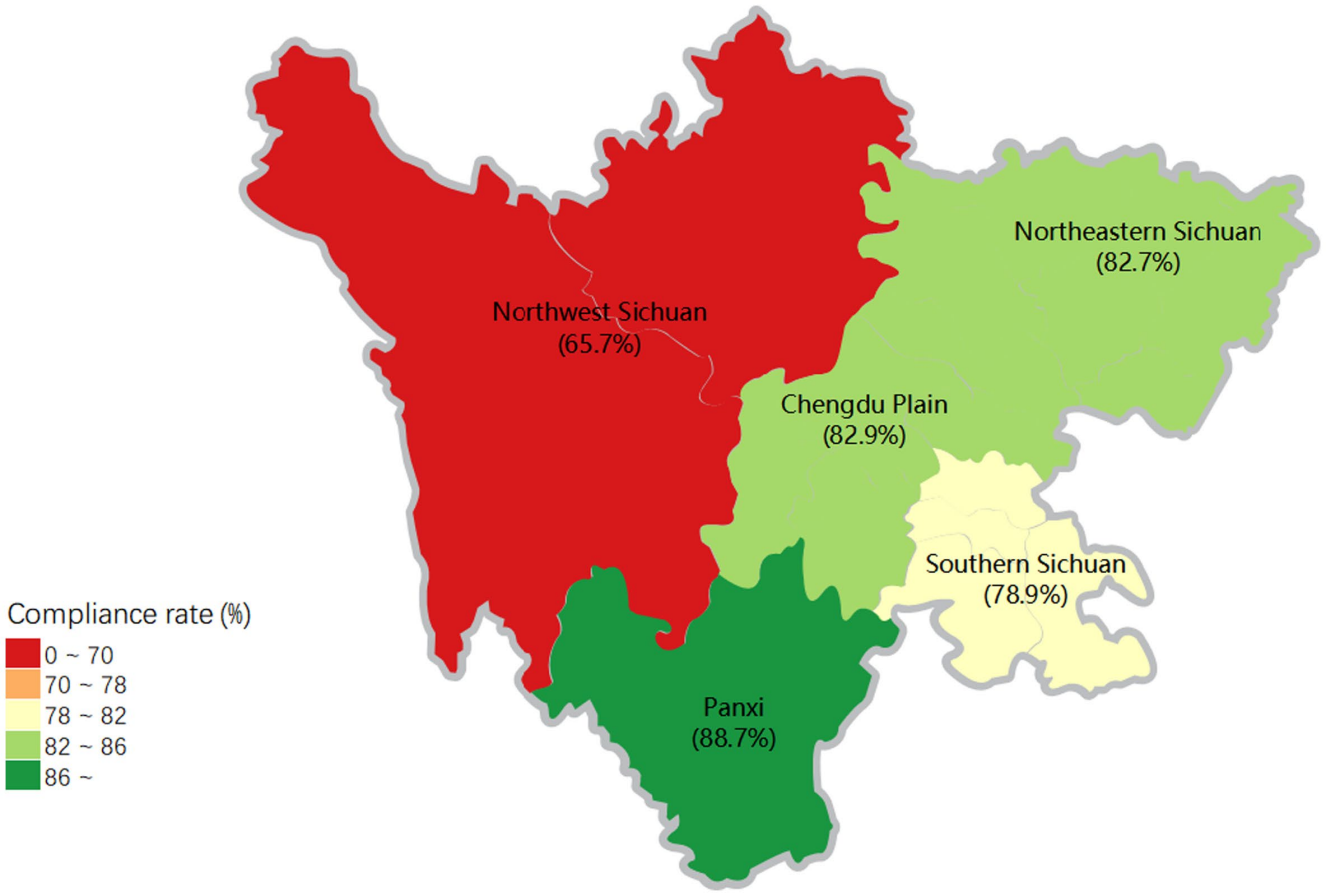
Regional compliance rate of occupational dust in Sichuan Province, 2021–2025.

- Enterprise scale disparity: A significant negative correlation was observed between enterprise scale and dust compliance rate (*χ*^2^ = 24.100, *p* < 0.05). Micro-enterprises achieved the highest compliance rate of 84.7%, while large-scale enterprises showed the lowest compliance rate at only 66.7%. The median dust concentration increased slightly with the expansion of enterprise scale, indicating that large enterprises generally have more complex production processes, which increases the difficulty of dust prevention and control.
- Regional disparity: Significant spatial heterogeneity in dust exposure was identified across the five economic zones of Sichuan Province (*p* < 0.001). The Northwest Sichuan Ecological Economic Zone exhibited the poorest dust control performance, with the lowest compliance rate of 65.7% and a median respirable dust concentration of 0.38 mg/m^3^. In contrast, the Panxi Economic Zone achieved the best control effect with a compliance rate of 88.7%. The spatial distribution characteristics of dust pollution risks are fully visualized in Figure 2.
- Industrial disparity: Obvious heterogeneity was observed across the four surveyed industries (*p* < 0.05). The machinery industry was the highest-risk sector, with the lowest compliance rate (75.9%) and the highest median dust concentration (0.36 mg/m^3^). The building materials industry ranked second in risk level with a compliance rate of 79.9%. The mining and metallurgy industries maintained relatively better control effects, with compliance rates of 85.0 and 86.8%, respectively.

### Independent predictors of respirable dust non-compliance (binary logistic regression analysis)

Binary logistic regression analysis was performed to screen independent influencing factors for dust compliance, with monitoring year, industry type, economic region, enterprise scale, and free silica content included in the model. The detailed regression coefficients, odds ratios, 95% confidence intervals, and *p*-values are shown in Table 3.

Time factor: Monitoring year was an independent protective factor for dust compliance. The compliance odds increased by 16.9% per year (*OR* = 1.169, 95%*CI*: 1.097–1.246, *p* < 0.05), verifying the continuous improvement of provincial dust control management level.
Industry factor: Taking the mining industry as the reference group, the metallurgy industry had a significantly higher compliance probability (*OR* = 1.774, *p* < 0.05). In contrast, building materials (*OR* = 0.682, *p* < 0.05) and machinery industries (*OR* = 0.707, *p* = 0.009) had significantly lower compliance odds.
Regional factor: Compared with the Chengdu Plain (reference region), Southern Sichuan (*OR* = 0.714, *p* < 0.05), Panxi (*OR* = 0.590, *p* < 0.05), and Northwest Sichuan (*OR* = 0.249, *p* < 0.05) were independent risk factors for non-compliance. No significant difference was found in the Northeastern Sichuan region (*p* > 0.05).
Enterprise scale factor: Enterprise scale was negatively correlated with compliance probability. Compared with micro-enterprises, small, medium, and large enterprises had gradually decreased compliance odds (all *p* < 0.01), confirming the adverse correlation between enterprise scale and dust control effect in Sichuan enterprises.
Free silica content factor: Free silica content was a significant risk factor for non-compliance. For every 1% increase in free silica content, the probability of dust compliance decreased by 2.5% (*OR* = 0.975, 95%*CI*: 0.971–0.979, *p* < 0.05).

**Table 3:** Logistic regression analysis on influencing factors of respirable dust compliance rate.

Variable	Category	*β*	SE	OR	95% CI	*P-*value
Year (continuous)	—	0.156	0.033	1.169	1.097—1.246	<0.001
Industry sector (ref = mining)	Metallurgy	0.573	0.145	1.774	1.334—2.36	<0.001
	Building materials	−0.382	0.134	0.682	0.525—0.887	0.004
	Machinery	−0.346	0.132	0.707	0.546—0.917	0.009
Economic region (ref = Chengdu Plain)	Southern Sichuan	−0.337	0.135	0.714	0.548—0.931	0.013
	Panxi	−0.528	0.153	0.59	0.437—0.796	<0.001
	Northeastern Sichuan	0.272	0.152	1.313	0.975—1.769	0.073
	Northwest Sichuan	−1.389	0.174	0.249	0.177—0.351	<0.001
Enterprise size (ref = micro)	Small	−0.287	0.109	0.751	0.606—0.93	0.009
	Medium	−0.602	0.164	0.548	0.397—0.755	<0.001
	Large	−0.986	0.312	0.373	0.203—0.687	0.002
Free SiO₂ content (%)	—	−0.026	0.002	0.975	0.971—0.979	<0.001

*β*, regression coefficient; SE, standard error; OR, odds ratio; 95% CI, 95% confidence interval; Ref, reference category.

### Industry- and region-specific stratified trend analysis

Further stratified analysis by industry and region verified interactive risk differences, and the detailed stratified results of annual compliance trends and regional differences for each industry are presented in Table 4. Both the mining and metallurgy industries achieved significant annual improvement in dust compliance during the study period (all *p* < 0.001). However, high-risk sub-groups still existed within these two industries: large-scale mining enterprises and mining posts with high free silica content had sharply reduced compliance rates. The machinery industry showed the most prominent risk characteristics, with compliance rates decreasing significantly as enterprise scale increased (*χ*^2^ = 37.248, *p* < 0.001), the compliance rate of large machinery enterprises dropped to 61.5%. Although the building materials industry showed a slight improving trend over years, it faced severe regional imbalance, with the compliance rate in the Northwest Sichuan region falling to 50%.

**Table 4:** Stratified analysis of silica dust monitoring results in key industry sectors in Sichuan Province, 2021–2025: interaction effects.

Characteristic/category	Mining		Metallurgy		Building materials		Machinery	
	No. of posts monitored	Compliance rate (%)	No. of posts monitored	Compliance rate (%)	No. of posts monitored	Compliance rate (%)	No. of posts monitored	Compliance rate (%)
Year								
2021	261	72	203	77.8	168	79.2	207	73.9
2022	215	85.6	196	91.8	132	88.6	186	71
2023	207	79.2	196	85.2	146	78.8	171	82.5
2024	204	93.6	201	86.1	118	84.7	174	71.3
2025	212	92.9	202	93.6	117	71.8	153	82.4
*χ*_trend_^2^ value		44.141		11.923		2.153		2.58
*P-*value		<0.001		<0.001		0.142		0.108
Economic zone								
Chengdu Plain	288	89.2	525	92.2	231	77.1	659	74.7
Southern Sichuan	280	77.9	15	100	202	81.2	101	74.3
Northeastern Sichuan	336	89	57	93	201	87.6	88	87.5
Panxi	150	81.3	238	84.9	33	72.7	35	82.9
Northwest Sichuan	45	62.2	163	69.3	14	50	8	37.5
*χ*^2^*-*value		36.768		62		17.834		14.54
*P-*value		<0.001		<0.001		0.001		0.006
Enterprise scale								
Micro	502	83.9	134	88.8	376	83.8	204	85.8
Small	506	85.0	715	87.0	277	76.5	549	77.4
Medium	74	87.8	131	85.5	19	73.7	125	54.4
Large	17	47.1	18	77.8	9	88.9	13	61.5
*χ*_trend_^2^ value		1.134		1.534		3.341		37.248
*P-*value		0.287		0.216		0.068		<0.001

Regionally, the Northwest Sichuan region was the high-risk area for all industries. Particularly, the machinery industry in Northwest Sichuan had an extremely low compliance rate of 37.5%, far lower than the provincial average level, representing the key weak link in regional occupational dust prevention and control. Notably, individual small-sample subgroups with extreme concentration values (e.g., machinery posts in Northwest Sichuan) were interpreted cautiously in this study due to limited sample size, avoiding overinterpretation of statistical results.

## Discussion

### Temporal trends and policy implications

The observed upward trend in compliance rates from 2021 to 2025 reflects the positive impact of strengthened regulatory enforcement and the implementation of special governance actions in Sichuan Province. This aligns with global observations where policy-driven interventions, such as mandatory hazard declaration and intensified inspections, have led to measurable improvements in occupational hygiene (18, 19). However, the persistence of an 18% non-compliance rate indicates that a significant portion of the workforce remains at risk. Notably, the lack of a corresponding downward trend in median respirable dust concentrations suggests that while more posts are meeting the threshold, the absolute reduction in dust load may be insufficient to eliminate long-term health risks, particularly given evidence that low-level chronic exposure still contributes to silicosis incidence (20).

### Unconventional scale patterns and industrial specificity

A critical finding of this study is the inverse relationship between enterprise scale and compliance, challenging the conventional wisdom that larger enterprises possess superior management capabilities. This “Unconventional Scale Patterns” is particularly evident in the Machinery and Mining sectors. Large enterprises often involve complex, multi-stage production processes where dust control systems may fail to keep pace with capacity expansions or technological upgrades (21). In contrast, some micro-enterprises with simpler processes may achieve compliance through basic wet methods or local exhaust ventilation, albeit potentially lacking systematic management (22). The Machinery industry emerged as the highest-risk sector, characterized by dispersed dust generation points during casting and grinding. This finding underscores the need for sector-specific engineering solutions, as traditional centralized dust collection systems may be ineffective for open or semi-open operations typical in machinery manufacturing (23). Similarly, the Building Materials sector’s struggles, especially with high-silica stone processing, highlight the urgent need for substituting high-silica materials or adopting advanced wet-cutting technologies (24).

Furthermore, the three non-compliance predictors identified in this study—enterprise scale, geographical region, and free silica content—are highly consistent with the findings of the latest U.S. research on silica dust in mines (25). The only difference is that high risks in the U.S. are concentrated in metal/nonmetal mines, while China’s Sichuan machinery industry, characterized by dispersed dust generation during casting and grinding processes, stands out as an independent highest-risk sector.

### Impact of free silica content and regional disparities

The strong negative correlation between free silica content and compliance reinforces the toxicological reality that high-silica dust is inherently more difficult to control due to its physical properties and stricter exposure limits (26). Posts with >50% free silica content require more sophisticated engineering controls, which are often economically or technically challenging for many enterprises to implement and maintain (27). Geographically, the Northwest Sichuan Ecological Economic Zone represents a critical vulnerability. As an underdeveloped region, it likely suffers from weaker regulatory oversight, older equipment, and a concentration of small-scale mining and processing activities (28). This mirrors global patterns where remote and economically disadvantaged regions bear a disproportionate burden of occupational diseases (29). Targeted support, including technical assistance and subsidized upgrades for enterprises in this region, is essential to bridge the gap (30).

### Limitations

This study has several limitations. First, the data are derived from declared enterprises, potentially excluding unregistered or informal operations where exposure levels might be even higher (31). Therefore, the findings can only be generalized to formally operating enterprises. Second, no individual-level exposure and PPE compliance data were collected, limiting assessment of actual worker exposure. Finally, the cross-sectional nature of the annual snapshots limits causal inferences regarding specific interventions.

## Conclusion

From 2021 to 2025, Sichuan Province achieved notable progress in the prevention and control of silica dust hazards, with a steady improvement in compliance rates. Nevertheless, prominent challenges persist in silica dust management. Weak links still lie in the machinery and building materials industries, large-scale enterprises with complex production processes, workplaces with high free silica content, and the northwestern region of Sichuan. To achieve the goal of eliminating silicosis, future strategies must shift from broad-spectrum regulation to targeted risk intervention:

1. Technological innovation: Mandate and subsidize the adoption of advanced dust control technologies (e.g., high-efficiency filtration, automated closed-loop systems) specifically for high-silica and machinery sectors.
2. Differentiated regulation: Implement risk-based grading where large, complex enterprises and those in underdeveloped regions receive heightened scrutiny and technical support.
3. Source substitution: Accelerate the promotion of low-silica alternative materials in the building materials industry.
4. Integrated surveillance: Strengthen the linkage between environmental monitoring and worker health surveillance to enable early detection and intervention.

By addressing these specific vulnerabilities, Sichuan can move towards a more effective and equitable occupational health system, ultimately protecting the respiratory health of its workforce.

## Data Availability

The datasets presented in this article are not readily available because they contain sensitive information regarding the occupational health of workers. Access to the data is restricted to protect the privacy and confidentiality of the participants. Requests to access the datasets should be directed to the corresponding author. Requests to access these datasets should be directed to WC, 44415630@qq.com.
